# Towards adaptive radiotherapy: Comparing HyperSight and standard CBCT reconstructions on a C‐Arm Linac

**DOI:** 10.1002/acm2.70786

**Published:** 2026-09-10

**Authors:** Niamh L. Clarke, Jeremy L. Hughes, Derrick Wanigaratne, Adam U. Yeo

**Affiliations:** ^1^ Department of Radiation Oncology Peter MacCallum Cancer Centre Melbourne Victoria Australia; ^2^ Sir Peter MacCallum Department of Oncology The University of Melbourne Melbourne Victoria Australia; ^3^ School of Applied Science RMIT University Melbourne Victoria Australia

**Keywords:** adaptive radiation therapy, advanced imaging, IGRT, HyperSight

## Abstract

**Background:**

Cone‐beam computed tomography (CBCT) is integral to image‐guided radiation therapy and increasingly used for adaptive radiotherapy and CBCT‐based dose calculation. Conventional kV‐CBCT systems on C‐arm TrueBeam linacs with Feldkamp–Davis–Kress (FDK) reconstruction have historically been limited by reduced Hounsfield Unit (HU) accuracy, scatter contamination, image noise and reconstruction artefacts. Iterative CBCT (iCBCT) reconstruction has improved image quality and HU consistency relative to the conventional FDK reconstruction. HyperSight represents a further evolution through software and hardware development with an enhanced detector design, advanced reconstruction algorithms and gantry rotation speeds up to 9°/s.

**Purpose:**

This study reports commissioning and evaluation of HyperSight iterative CBCT (HS‐iCBCT) acquired on a Varian TrueBeam (v4.1), to support adaptive workflows in comparison with standard (Std‐FDK) and iterative CBCT (Std‐iCBCT) on a TrueBeam (v2.7).

**Material and methods:**

CBCT images were acquired on a Varian TrueBeam using the conventional kV On‐board Imager (OBI) for Std‐FDK and Std‐iCBCT reconstructions and the HyperSight kV‐imaging system for HS‐iCBCT. Commissioning included CBCT dose calculation protocol optimization, CT‐to‐electron density (CT–ED) calibration and validation using STEEV, Rando and the CIRS Dynamic Thorax phantoms. CBCT images of anthropomorphic Head, Thorax/Spine, Rib and Lung phantoms were rigidly registered to the planning CT (pCT) and resampled using Velocity AI (v4.2). Voxel‐wise HU difference histograms were generated, with peak position used to quantify systematic HU bias. Treatment plans (20 Gy/1# for Brain SRS/Spine, 24 Gy/2# for Rib and 54 Gy/3# for Lung) were optimized on the pCT and recalculated on the CBCT datasets. Dose–volume histogram (DVH) metrics were assessed and 3D‐gamma analysis (2%/1 mm to 1%/1 mm; 10%/70% dose thresholds) was performed and compared with Std‐FDK and Std‐iCBCT.

**Results:**

HS‐iCBCT showed improved HU accuracy compared with Std‐iCBCT and Std‐FDK when benchmarked against the pCT. For materials ≤ 1.08 g/cm^3^, the mean HU difference was lowest for HS‐iCBCT at 7.7 ± 4.1 HU, compared with 23.6 ± 13.5 HU for Std‐iCBCT and 38.0 ± 19.7 HU for Std‐FDK (Friedman *p* < 0.05). For higher‐density materials, HS‐iCBCT demonstrated mean HU differences of 33.5 ± 22.9 HU, compared with 46.2 ± 22.2 HU for Std‐iCBCT and 93.4 ± 57.6 HU for Std‐FDK. HS‐iCBCT improved image quality with HU difference histograms demonstrating peak values closest to zero (∆HU within 15HU). An optimized CBCT acquisition protocol (140 kV) combined with a single baseline 120 kV pCT HU–ED calibration curve proved feasible for CBCT‐based dose calculation. Dose calculation agreement was strongest for HS‐iCBCT (*p* < 0.05) with median target dose differences measuring < 0.3%, < 0.6%, 0.6% and < 0.4% for the Brain, Thorax/Spine, Rib, and Lung anatomies, respectively. Median (min‐max) gamma passing rates across all anatomical sites were 98.8% (97.8–99.8), 98.7% (96.7–99.6), and 96.0% (94.5–99.5) with 2%/1 mm passing criteria for HS‐iCBCT, Std‐iCBCT, and Std‐FDK, respectively.

**Conclusions:**

HyperSight reduces HU variability, improves image quality, and enhances CBCT‐based dose calculation relative to conventional CBCT implementations. Use of a single optimized CBCT dose calculation protocol and baseline HU–ED calibration curve proved feasible across multiple anatomical sites, supporting implementation of HS‐iCBCT for adaptive radiotherapy workflows on conventional C‐arm TrueBeam linacs.

## 1 INTRODUCTION

Cone‐beam CT (CBCT) is widely used in image‐guided radiotherapy (IGRT) to verify patient setup and tumor position before treatment.^1^ Standard kV on‐board imagers (OBI) on Varian TrueBeam linacs typically use the Feldkamp–Davis–Kress (FDK) reconstruction algorithm, which generates three‐dimensional (3D) images from two dimensional (2D) cone‐beam projections using one‐dimensional (1D) filtering, geometric weighting, and back‐projection.^2^

Although efficient and clinically practical, FDK‐CBCT is susceptible to scatter, noise, beam hardening, and truncation artefacts. These limitations reduce low‐contrast visibility and compromise Hounsfield Unit (HU) accuracy, rendering standard CBCT inferior to conventional fan‐beam geometries used for planning CTs (pCT).

Iterative CBCT (iCBCT) reconstruction was developed to overcome the long‐standing limitations of conventional FDK‐based imaging on TrueBeam linacs. It employs the Acuros CT scatter (CTS) algorithm alongside a statistical model that enforces projection data fidelity while accounting for system geometry, detector response, noise statistics and regularization constraints.^3^, ^4^ These model‐based features improve soft tissue contrast and HU accuracy, extending the role of CBCT beyond patient positioning. Multiple studies have confirmed these benefits, reporting improved contrast to noise ratio (CNR), reduced noise and image artefacts and better HU stability, all relevant to the use of CBCT for dose calculation and adaptive planning.^5^, ^6^, ^7^, ^8^

HU accuracy directly underpins dose calculation accuracy and different approaches can be used when performing calculations on CBCT datasets to characterize the relationship between the HU and electron densities (ED) in patient anatomy on‐treatment. These include the use of pCT‐derived HU‐ED calibration curve, deformable registration and site specific CBCT calibration curves, among others.^9^ Lim et al assessed whether CBCT can reliably use pCT‐based HU‐ED curves or required protocol‐specific calibration through systematically comparing multiple CBCT acquisition parameters (varying kV, mAs and FOV) and reconstruction methods (FDK vs. iCBCT).^5^ Their study demonstrated that protocol choice is a dominant factor in HU fidelity in addition to reconstruction method. In particular, protocols employing higher kV and mAs settings together with larger FOVs, when coupled with iterative reconstruction, showed substantially improved agreement with baseline pCT values. These findings indicated that iCBCT may support dose calculation using standard pCT HU‐ED curves without the need for protocol‐specific CBCT calibration.

Hardware advances have further improved CBCT performance. HyperSight was initially developed for O‐ring linac systems such as Halcyon and Ethos, where improved detector design, faster gantry rotation, mechanical stability and advanced GPU‐based reconstruction support better soft‐tissue visibility, HU accuracy and online adaptive radiotherapy (oART), workflows. However, investment in such O‐ring systems remains substantial, motivating interest in improving conventional C‐arm platforms such as TrueBeam linacs to achieve comparable image quality improvements.

HyperSight has recently been extended to TrueBeam linacs, with hardware and software upgrades intended to improve image quality over standard FDK, (Std‐FDK), and iterative CBCT, (Std‐iCBCT), narrowing the historical image‐quality gap between C‐arm and O‐ring platforms. These include a higher scatter grid ratio, 15:1 to reduce scatter artefacts, a larger 43 × 43 cm^2^ detector, extended field‐of‐view (FOV) reconstruction up to 70 cm, faster gantry acquisition of 9°/s, enhanced image processing and iterative metal artefact reduction reconstruction, iCBCT MAR.

Most HyperSight studies have focused on O‐ring systems, demonstrating improved image quality, target and OAR visualization, HU accuracy and CBCT‐based adaptive dose calculation.^10^, ^11^, ^12^, ^13^, ^14^ Limited evidence exists for HyperSight on conventional TrueBeam linacs, and to date, no study has directly compared HyperSight iterative CBCT on TrueBeam v4.1, HS‐iCBCT, with Std‐FDK and Std‐iCBCT on TrueBeam v2.7. Addressing this gap is particularly relevant for centers where major hardware investment may not be feasible. Accordingly, this study evaluates HS‐iCBCT on TB4.1 against Std‐FDK and Std‐iCBCT on TB2.7 to assess differences in image quality, HU accuracy, dose calculation suitability and adaptive workflow efficiency.

## 2 MATERIALS AND METHODS

### 2.1 Image acquisition

In this study, CBCT images were acquired using the novel HyperSight CBCT imaging system on a TrueBeam v4.1 platform and compared with a previous‐generation TrueBeam v2.7 system equipped with the standard OBI kV platform and iCBCT reconstruction. The HyperSight system features a larger detector panel (43 × 43 cm^2^) and a faster gantry rotation speed (9°/s; 1.5 rpm). In contrast, the standard OBI system employs a smaller detector panel (40 × 30 cm^2^) and operates at a slower gantry speed (6°/s; 1 rpm).

On the HyperSight system, CBCT datasets were reconstructed using the iCBCT reconstruction only. For the standard TrueBeam system, two reconstruction methods were utilized: The conventional FDK reconstruction and its updated implementation, iCBCT. As both TrueBeam systems were capable of iCBCT reconstruction, datasets from the HyperSight system are hereafter referred to as HS‐iCBCT, while those from the standard on‐board kV imaging system are denoted as Std‐iCBCT.

### 2.2 Anthropomorphic phantoms

Three primary anthropomorphic phantoms were used in this study for the evaluation of CBCT‐based dose calculation. These comprised of the STEEV phantom (Sun Nuclear Corporation, Melbourne, FL, USA) to simulate head and brain anatomy, the RANDO phantom (Alderson RANDO, Alderson Research Laboratories Inc., Long Island City, NY, USA) for abdominal and bony rib anatomies, and a dynamic CIRS phantom (Sun Nuclear Corporation) to represent spine and lung anatomy. The CIRS Dynamic Thorax phantom was scanned under static acquisition conditions, representing a breath‐hold or motion‐managed treatment scenario. To establish a baseline, all phantoms were scanned on the Philips Big Bore CT simulator (Philips Medical Systems, Cleveland, USA) using the default institutional protocol for that site at 140 kV.

### 2.3 HU‐ED calibration curve and CBCT protocol optimization

The Advanced Electron Density Phantom (Model 1467, Sun Nuclear Corporation, Melbourne, FL, USA) was used to generate Hounsfield unit–electron density (HU–ED) calibration curves and enable quantitative assessment of dose calculation accuracy using CBCT images. The phantom comprised of two sections: an inner section with a diameter of 20 cm and an outer section with a diameter of 40 cm, representing head and body geometries, respectively. The phantom contained inserts spanning a range of known electron densities up to dense bone (*ρ* ≤ 1.9 g/cm^3^). Reference scans were acquired using a Philips Brilliance Big Bore CT simulator acquired using our institution's standard pCT protocol (140 kV), and an additional 120 kV acquisition, with both datasets reconstructed using a quantitative (Qr40) kernel. All images were reconstructed with a slice thickness of 2 mm.

Separate HU–ED calibration curves were generated for each TrueBeam imaging system using iCBCT reconstruction. For the standard on‐board kV imaging system, a calibration curve was additionally generated using FDK reconstruction for comparison. The resulting curves were defined as HU–ED_HS‐iCBCT_, HU–ED_Std‐iCBCT_ and HU–ED_Std‐FDK_, corresponding to HS‐iCBCT, Std‐iCBCT and Std‐FDK reconstructions.

Two in‐house optimized CBCT acquisition protocols (125 kV and 140 kV; 540–675 mAs; half‐fan geometry) were employed, adapted from the Varian Pelvis and Pelvis Large default CBCT protocols. Images were imported into the Eclipse treatment planning system (TPS) for analysis. Regions of interest (ROIs) were defined for each insert on the central plane, encompassing 70% of the phantom insert diameter over a longitudinal extent of 2 cm. The mean HU and standard deviation (SD) were extracted for each ROI and compared with baseline pCT HU values.

In total, six CBCT datasets were acquired: two for the HyperSight system reconstructed with iCBCT, and four for the standard system reconstructed using both iCBCT and FDK, as outlined in Table 1. Absolute HU differences (ΔHU) relative to the pCT baseline were subsequently calculated per insert and averaged across head and body geometries. Statistical differences between reconstruction methods (HS‐iCBCT, Std‐iCBCT and Std‐FDK) were assessed using a Friedman test. Where the Friedman test indicated a significant overall difference (*p* < 0.05), post‐hoc Nemenyi testing was performed for multiple comparisons.

**TABLE 1:** Planning CT (pCT) and cone‐beam CT (CBCT) image acquisition parameters for the advanced electron density phantom.

Modality	Reconstruction	Voltage (kV)	Exposure (mAs)	Geometry	Gantry rotation speed (°/s)	Slice thickness (mm)
pCT	Qr40	140	300	Full‐fan	—	2
		120	300	Full‐fan	—	2
HS‐CBCT	iCBCT	140	675	Half‐fan	9	2
		125	540	Half‐fan	9	2
Std‐CBCT	iCBCT / FDK	140	675	Half‐fan	6	2
		125	540	Half‐fan	6	2

### 2.4 Image quality and HU accuracy

All phantoms were scanned on each TrueBeam using the CBCT protocols outlined in Table 1. In addition to the clinical 140 kV protocol, an additional 100 kV acquisition (100mAs, half‐fan) was performed to assess the effect of beam energy on image quality and dose calculation accuracy. For each phantom, a total of six CBCT datasets were acquired, representing combinations of different hardware configurations, software implementations, and reconstruction algorithms.

CBCT images were rigidly registered to the planning CT (pCT) and resampled using Velocity AI (v4.2) to ensure the CBCT and pCT images shared the same DICOM frame of reference. To assess image quality, voxel‐wise signed HU difference histograms were generated for each CBCT acquisition where ∆HU was defined as the voxel‐wise difference between the pCT and CBCT (∆HU = HU_pCT_—HU_CBCT_). The peak position and the full width half maximum (FWHM) characterizes the systematic HU offset and the spread of HU differences relative to the pCT. In the case of perfect agreement between the CBCT and pCT, the HU difference distribution would converge to a Dirac delta function centered at 0 HU. Deviations from this ideal distribution therefore quantify energy and reconstruction‐dependent HU discrepancies.

A qualitative comparison of image quality was performed for patients undergoing daily CBCT imaging. Offline reconstructions using HS‐FDK and HS‐iCBCT algorithms were generated across multiple clinical sites, including the Brain, pelvis, and thorax, to evaluate differences in image quality between the reconstruction methods.

### 2.5 Treatment planning and dosimetric accuracy

For each anthropomorphic phantom, pCT scans were imported into Eclipse TPS. A total of four treatment plans on each phantom were created using the; (1) Original pCT, (2) HS‐iCBCT, (3) Std‐iCBCT and (4) Std‐FDK dataset for each CBCT protocol at 140 kV. For each CBCT plan, the CT baseline HU‐ED_120kV_ calibration curve was applied for dose calculation. Planning target volume (PTV) expansions and organs at risk (OARs) were delineated on the pCT images and subsequently propagated to the resampled CBCT images.

**FIGURE 1:**
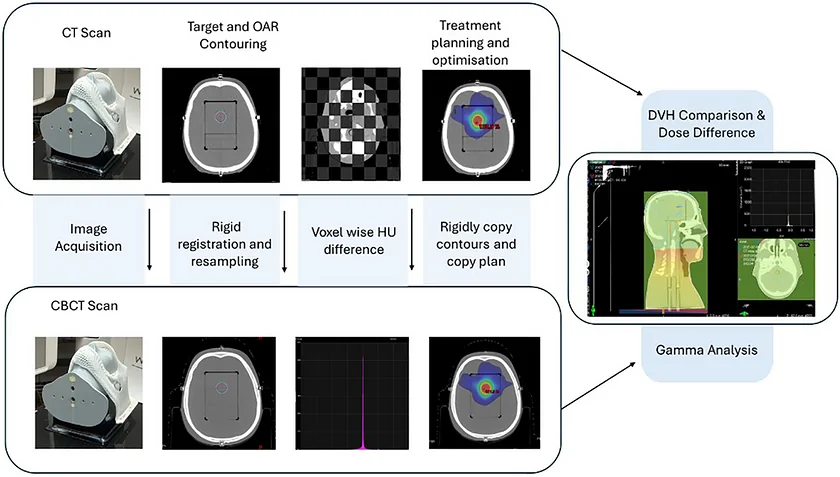
Overview of treatment planning and dose calculation comparison between pCT and CBCT datasets. Following rigid registration and image resampling in Velocity AI (v4.2), absolute HU differences were calculated. Contours and treatment plans were transferred from pCT to CBCT and recalculated using identical treatment parameters prior to DVH and gamma analysis for dose comparison.

For the STEEV phantom, a stereotactic radiosurgery (SRS) plan containing a single intracranial metastasis was created with 20 Gy/1#. For the CIRS lung phantom, a stereotactic ablative radiotherapy (SABR) plan delivering 54 Gy/3# was generated to a single central mass and an additional spine SABR plan delivering 20 Gy/1# was also created. For the Rando phantom, a SABR Rib 24 Gy/2# plan was generated. These plans were selected to represent a range of clinically relevant treatment sites and differing levels of geometric and dosimetric complexity. The original pCT which was calculated using the AXB dose calculation algorithm (v18) was copied to the CBCT dataset and recalculated using the same monitor units (MUs), beam geometries, and calculation settings as illustrated in Figure 1.

Dose‐volume histogram (DVH) metrics for the PTVs and relevant OARs were extracted to enable dosimetric comparison between the pCT and CBCT images, with the pCT considered the gold‐standard throughout. For target volumes, D98% or D99%, D50%, D2% and D0.03cc were reported. For OARs, the near maximum (D_0.03cc_) or closest relevant metric was evaluated. Three‐dimensional (3D) gamma analysis was performed in Python v3.12 using the pymedphys (v0.41.0) library for comparison of dose distributions between the pCT and CBCT‐based plans. Gamma criteria ranging from 2%/1 mm to 1%/1 mm were applied, with dose thresholds of 10% and 70% used to assess agreement in low‐ and high‐dose regions, respectively. Statistical analysis was performed using the Friedman test to assess differences in dosimetric parameters and gamma pass rates among the HS‐iCBCT, Std‐iCBCT, and Std‐FDK plans. A *p*‐value < 0.05 was considered statistically significant. Post‐hoc pairwise comparisons were performed only following a significant Friedman test.

## 3 RESULTS

### 3.1 HU‐ED characterization

**FIGURE 2:**
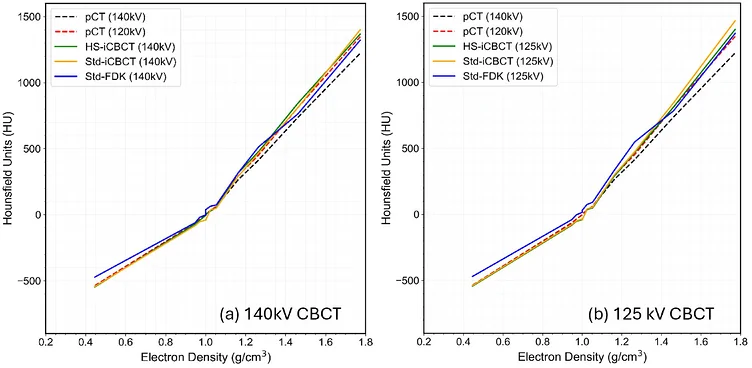
HU–electron density (HU–ED) calibration curves for CBCT reconstructions acquired using (a): 140 kV and (b) 125 kV CBCT protocols averaged across head and body geometries. CBCT‐derived HU values for HS‐iCBCT, Std‐iCBCT, and Std‐FDK are compared with reference planning CT (pCT) datasets acquired at 120 and 140 kV.

**FIGURE 3:**
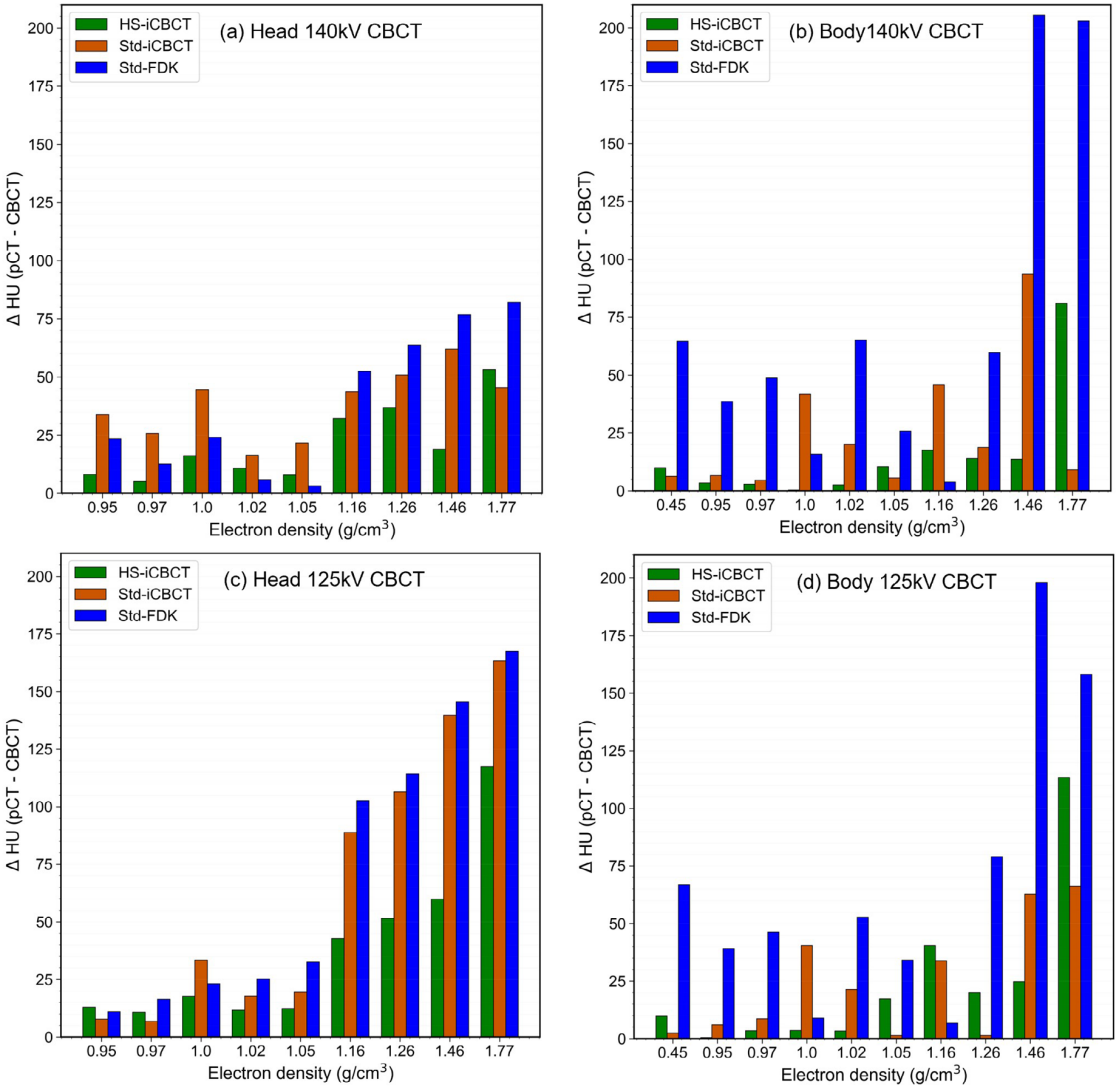
Absolute HU difference values across inserts in the SNC phantom relative to the pCT (120 kV) for head and body geometries across FDK and iCBCT reconstructions (a) Head 140 kV (b) Body 140 kV (c) Head 125 kV and (d) Body 125 kV.

The HU values from the CBCT images across the HyperSight and conventional kV imaging systems were compared to the baseline pCT HU‐ED curve for both 140 and 120 kV. Figure 2 demonstrates that 140 kV pCT consistently exhibited larger deviations from CBCT measurements across all reconstruction methods (HS‐iCBCT, Std‐iCBCT, and Std‐FDK) and for both CBCT acquisition protocols (125 and 140 kV) averaged across head and body geometries. This effect was most pronounced at higher electron densities, where increased ΔHU across all reconstructions was seen (Figure 3). In contrast, the 120 kV pCT showed improved agreement with CBCT‐derived HU values across the full density range, with reduced systematic offsets. Consequently, the 140 kV pCT was deemed inferior for HU–ED calibration and the 120 kV pCT was selected as the reference standard for subsequent analysis.

### 3.2 Optimization and selection of CBCT dose calculation protocol

The absolute difference for each insert for both 125 and 140 kV acquired CBCT protocols was calculated for each reconstruction method. Figure 3 shows the differences for (a) Head and (b) Body geometries at 140 kV and (c) Head and (d) Body geometries at 125 kV. Overall, HU deviations for the 140 kV CBCT protocol were smaller relative to the baseline pCT compared to the 125 kV CBCT protocol. At 140 kV acquisition settings, the average HU difference for water‐equivalent materials (≤ 1.08 g/cm^3^) across head and body phantoms was 38.0 ± 19.7 HU for Std‐FDK, 23.6 ± 13.5 HU for Std‐iCBCT and 7.7 ± 4.1 HU for HS‐iCBCT. For 125 kV acquisition settings, deviations were generally higher, with average HU differences at water equivalent materials 36.8 ± 15.2 for Std‐FDK, 18.0 ± 14.6 for iCBCT and 9.4 ± 3.9 HU for HS‐iCBCT.

At higher densities (1.08 g/cm^3 ^< *ρ* ≤ 1.92 g/cm^3^), Std‐FDK varied the most across both acquisition energies, particularly for cortical bone. At 140 kV, the average difference was 93.4 ± 57.6 HU for Std‐FDK, 46.2 ± 22.2 HU for Std‐iCBCT and 33.5 ± 22.9 for HS‐iCBCT. At 140 kV, maximum differences averaged across head and body geometries were 142.6 ± 85.4 HU for Std‐FDK for cortical bone, 77.8 ± 22.3 HU for Std‐iCBCT for CaCO_3_50% and 67.2 ± 19.7 HU for HS‐iCBCT for cortical bone. At 125 kV, average HU differences were 102.1 ± 44.8 HU for Std‐FDK, 82.8 ± 29.8 HU for Std‐iCBCT and 24.2 ± 37.8 for HS‐iCBCT with the largest differences of 162.8 ± 72.2 for Std‐FDK.

The 125 kV datasets demonstrated greater variability in HU values across inserts, consistent with increased sensitivity to scatter and acquisition‐related effects at lower beam energies. As a result, this protocol was deemed sub‐optimal for CBCT dose and subsequent analysis focused on the 140 kV CBCT acquisitions only, which provided more stable and reproducible HU measurements relative to the pCT reference.

A Friedman test demonstrated a statistically significant difference in ΔHU deviations across the three reconstruction methods (*p* < 0.05) at 140 kV acquisition settings. Post‐hoc Nemenyi analysis demonstrated lower ΔHU values for HS‐iCBCT relative to Std‐FDK (*p* < 0.05). HS‐iCBCT also demonstrated lower ΔHU values than Std‐iCBCT; however, the evidence following correction for multiple comparisons was less conclusive (*p *= 0.06). Likewise, the evidence for a difference between Std‐iCBCT and Std‐FDK was weaker (*p* = 0.23).

### 3.3 Phantom image quality

**FIGURE 4:**
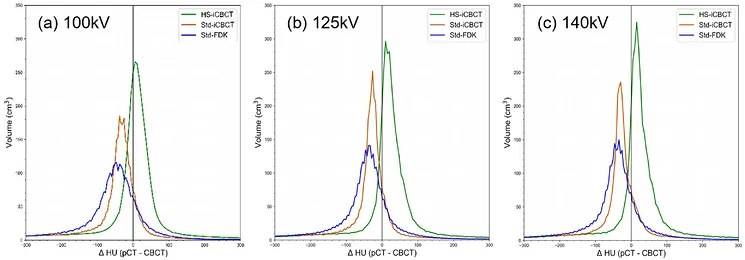
Voxel‐wise ΔHU (pCT − CBCT) histograms for HS‐iCBCT, Std‐iCBCT, and Std‐FDK reconstructions at (a) 100 kV, (b) 125 kV, and (c) 140 kV acquisitions for the STEEV phantom.

Figure 4 shows the voxel‐wise ΔHU histograms for the STEEV phantom to demonstrate the effect of beam energy and reconstruction on HU accuracy. In general, the voxel‐wise ΔHU histograms are all symmetrically distributed around the peak ΔHU. It's important to note the improved agreement with pCT with increasing tube potential across all reconstruction methods.

Histogram analysis demonstrated clear differences in HU accuracy and distribution width between reconstruction methods and acquisition energies (Table 2). In relation to pCT, Std‐FDK and Std‐iCBCT mostly overestimate HU (ΔHU < 0); whereas HS‐iCBCT tends to underestimate HU (ΔHU > 0). Increasing the acquisition energy resulted in a reduction in histogram width for all reconstruction methods. At 140 kV, further narrowing of the HU distribution was observed, with HS‑iCBCT achieving a peak ΔHU of +15 HU, compared with −30 HU for Std‑iCBCT and −34 HU for Std‑FDK. Across all acquisition energies, HS‑iCBCT consistently yielded peak HU values closest to zero, indicating reduced HU bias, while maintaining substantially narrower HU distributions on average. Optimal HS‐iCBCT performance was achieved using a 140 kV acquisition, demonstrating the most favorable balance between HU accuracy and noise reduction.

**TABLE 2:** Peak ΔHU and full width at half maximum (FWHM) of voxel‐wise ΔHU distributions for each phantom, reconstruction method, and CBCT acquisition energy. Peak ΔHU represents systematic HU bias relative to pCT (120 kV), while FWHM characterizes distribution spread.

Anthropomorphic Phantom	CBCT Acquisition Energy (kV)	Imaging System/ Reconstruction	Peak ΔHU	FWHM (ΔHU)
STEEV	100	HS‐iCBCT	+6	55
		Std‐iCBCT	−38	41
		Std‐FDK	−47	94
	125	HS‐iCBCT	+10	50
		Std‐iCBCT	−27	35
		Std‐FDK	−46	41
	140	HS‐iCBCT	+15	39
		Std‐iCBCT	−30	31
		Std‐FDK	−34	62
Rando	140	HS‐iCBCT	+12	33
		Std‐iCBCT	+25	45
		Std‐FDK	+8	68
CIRS Lung	140	HS‐iCBCT	+7	24
		Std‐iCBCT	+29	38
		Std‐FDK	+32	65

In the RANDO phantom, HS‐iCBCT demonstrated improved agreement with pCT, with a peak ΔHU of +12 HU compared to +25 HU and increased spread for Std‐iCBCT, while Std‐FDK showed reduced bias +8 HU but substantially greater variability (FWHM = 68 HU). Deviations were more pronounced in the CIRS lung phantom across all reconstruction methods, although HS‐iCBCT maintained comparatively lower bias (+7 HU) but with increased variability (FWHM = 24 HU) compared to Std‐iCBCT and Std‐FDK (+29 HU and +32 HU bias) and broad distributions (FWHM = 38–65 HU), demonstrating improved robustness in heterogeneous anatomy.

### 3.4 Patient image quality

**FIGURE 5:**
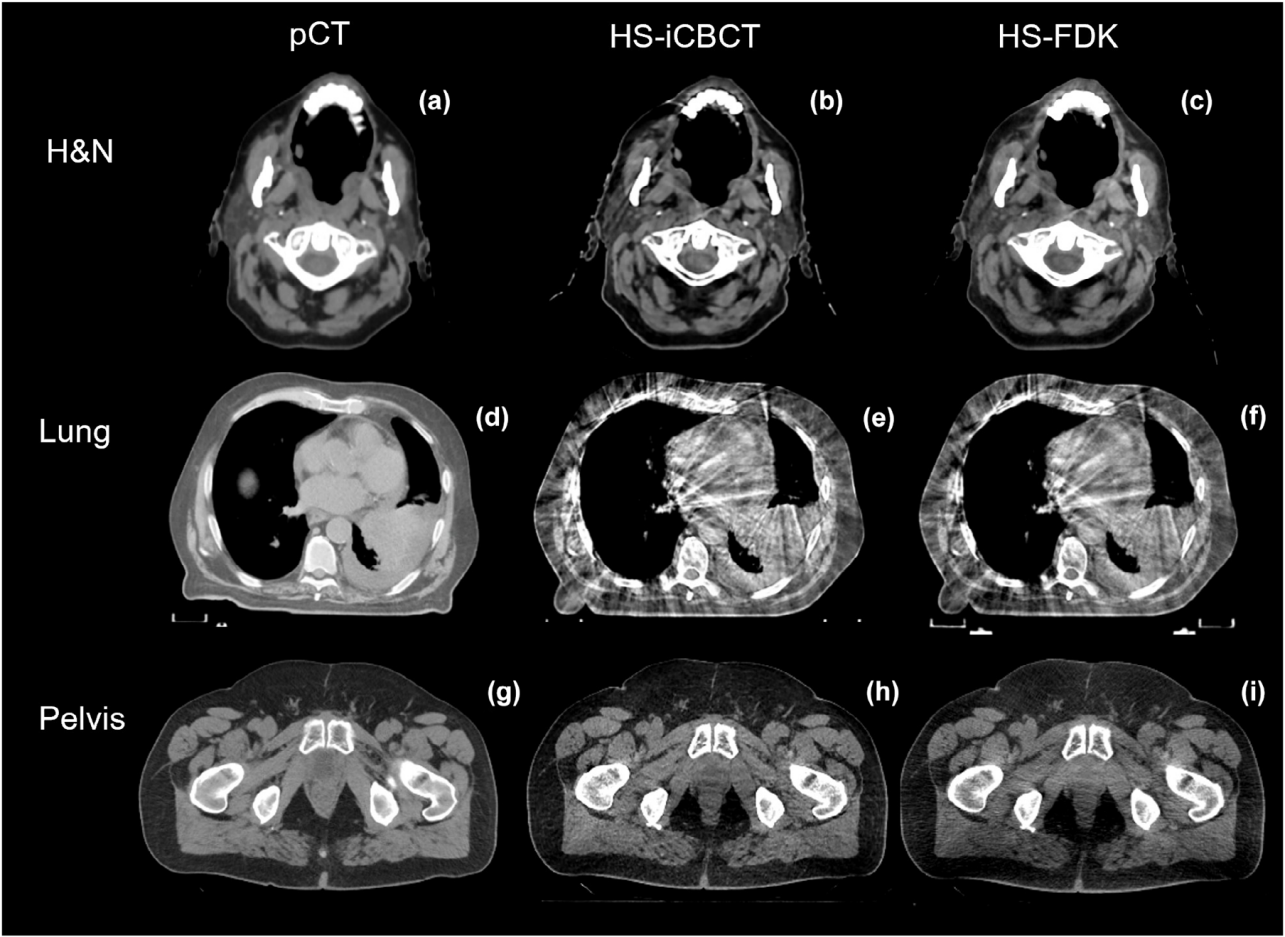
Patient image quality comparison between HS‐iCBCT and HS‐FDK reconstruction relative to the pCT for patient datasets acquired at a CBCT acquisition energy of 140 kV. (a–c) H&N patient, (d–f) Lung in free‐breathing and (g–i) Prostate patient.

Figure 5 displays axial patient images comparing the pCT, HS‐iCBCT and HS‐FDK CBCT reconstructions across H&N, lower thoracic and pelvic anatomies. Clear improvement can be seen in the HS‐iCBCT images compared to the same image reconstructed with FDK. In the pelvis and head‐and‐neck regions, HS‐iCBCT demonstrated enhanced uniformity and improved visualization of soft tissue structures, reduced noise and decreased streaking artefacts compared with FDK reconstruction. Motion artefacts remained visible in both HS‐iCBCT and HS‐FDK thoracic datasets, with persistent streaking and cupping artefacts associated with low‐density lung tissue and substantial respiratory motion (which is also applicable in Std‐iCBCT). While these artefacts may be further mitigated through motion management strategies such as respiratory gating, this was not evaluated in the present study. Nevertheless, low‐contrast soft tissue structures such as target and surrounding OARs (e.g. spinal cord) remained favorably matched with the HS‐iCBCT over HS‐FDK in thoracic images despite the presence of motion‐related degradation.

### 3.5 Dose calculation

**FIGURE 6:**
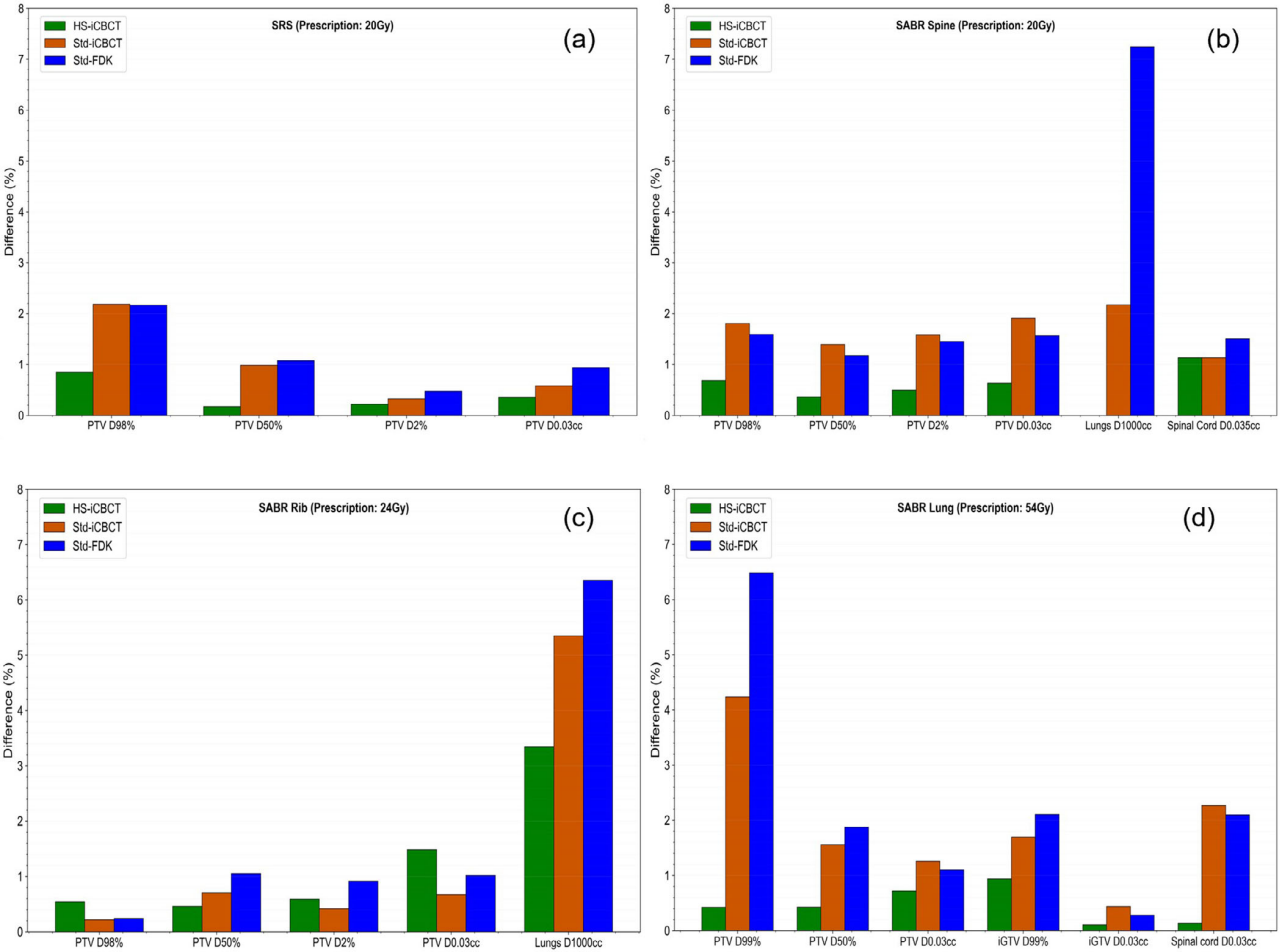
Absolute percentage differences in target and OAR DVH metrics relative to the planning CT (pCT) for (a) SRS (20 Gy/1fx), (b) SABR Spine (20 Gy/1fx), (c) SABR Rib (24 Gy/2fx), and (d) SABR Lung (54 Gy/3fx). Treatment plans were reconstructed using HS‐iCBCT, Std‐iCBCT, and Std‐FDK.

Figure 6 summarizes the absolute percentage differences in target and OAR DVH metrics relative to the pCT for each phantom/anatomy and reconstruction method. Across all anatomies, HS‐iCBCT demonstrated significantly lower target dose differences compared with the standard reconstruction methods (*p* < 0.05), with median deviations of < 0.3%, < 0.6%, < 0.6%, and < 0.4% for the Brain, Thorax/Spine, Rib, and Lung anatomies, respectively. In comparison, larger discrepancies were observed for Std‐iCBCT (0.8%, 1.7%, 0.5%, and < 1.6%) and Std‐FDK (1.0%, 1.5%, 1.0%, and 1.9%) across the corresponding anatomies for the Brain, Thorax/Spine, Rib, and Lung anatomies, respectively.

Larger deviations were observed for Std‐iCBCT and Std‐FDK in lung anatomies with an absolute dose deviation of 6.5% for PTV D99% using Std‐FDK, compared with 4.2% for Std‐iCBCT and 0.5% for HS‐iCBCT. In the SRS case, the maximum deviation relative to pCT was identified in PTVD98%, measuring 2.2% for Std‐FDK, 2.1% for Std‐iCBCT, and 0.8% for HS‐iCBCT. For the Spine SABR case, the largest percentage difference was observed in PTV D0.03cc using Std‐iCBCT (1.9%), with comparable values for Std‐FDK (1.6%) and lower differences for HS‐iCBCT (0.6%). The Rib case demonstrated similar agreement across all reconstruction methods within 0.8%, while the lungs metric (D1000cc) shows larger deviations of 6.4% for Std‐FDK and 5.4% for Std‐iCBCT, compared to 3.3% for HS‐iCBCT. Friedman testing demonstrated a statistically significant difference in dose deviations between the three reconstruction methods (*p* < 0.05). Post‐hoc Nemenyi analysis demonstrated significantly lower dose deviations for HS‐iCBCT relative to Std‐iCBCT and Std‐FDK (*p* < 0.05), while differences between Std‐iCBCT and Std‐FDK were not statistically significant (*p* = 0.18).

Gamma analysis results are presented in Table 3 for gamma criteria of 2%/1 mm and 1%/1 mm using 10% and 70% dose thresholds. HS‐iCBCT achieved the highest gamma pass rates across all criteria and dose thresholds, while Std‐FDK demonstrated the lowest agreement (*p* < 0.05). At 2%/1 mm and 10% dose threshold, median (min‐max) gamma pass rates across all anatomical sites were 98.8% (97.8−99.8), 98.7% (96.7−99.6), and 96.0% (94.5−99.5) for HS‐iCBCT, Std‐iCBCT, and Std‐FDK, respectively. Increasing the dose threshold to 70% amplified differences between reconstruction approaches, with pass rates of 97.1%–100% for HS‐iCBCT, 92.6%–96.4% for Std‐iCBCT and 83.6%–95.1% for Std‐FDK. The largest discrepancy was identified in the lung case, where Std‐FDK achieved a pass rate of 83.6% compared with 100% for HS‐iCBCT.

**TABLE 3:** Gamma pass rates for 10% and 70% dose thresholds at (a) 2%/1 mm and (b) 1%/1 mm criteria across different anatomical regions. Post‐hoc Nemenyi analysis demonstrated significantly higher gamma pass rates for HS‐iCBCT relative to Std‐FDK (*p* < 0.05), while differences between HS‐iCBCT and Std‐iCBCT and between Std‐iCBCT and Std‐FDK were not statistically significant.

(a) Anatomical Region	2%/1 mm (10% TH) HS‐iCBCT	2%/1 mm (70% TH) HS‐iCBCT	2%/1 mm (10% TH) Std‐iCBCT	2%/1 mm (70% TH) Std‐iCBCT	2%/1 mm (10% TH) Std‐FDK	2%/1 mm (70% TH) Std‐FDK
**SRS**	99.8	100.0	99.6	96.4	99.5	95.1
**Spine**	98.3	97.1	96.7	92.6	95.6	93.4
**Lung**	97.8	100.0	99.4	94.7	96.4	83.6
**Rib**	99.3	99.8	98.0	96.2	94.5	94.1

(b) Anatomical Region	1%/1 mm (10% TH) HS‐iCBCT	1%/1 mm (70% TH) HS‐iCBCT	1%/1 mm (10% TH) Std‐iCBCT	1%/1 mm (70% TH) Std‐iCBCT	1%/1 mm (10% TH) Std‐FDK	1%/1 mm (70% TH) Std‐FDK
**SRS**	99.6	97.7	99.3	91.9	99.0	87.9
**Spine**	97.2	94.6	93.6	86.6	92.0	88.6
**Lung**	96.8	100.0	98.1	89.3	94.5	77.7
**Rib**	98.7	98.9	97.0	93.9	93.5	91.6

## 4 DISCUSSION

HyperSight CBCT imaging has the potential to support adaptive radiotherapy planning and re‐planning workflows through improved image quality and HU accuracy. This study evaluated HS‐iCBCT acquired on a TrueBeam v4.1 platform for adaptive radiotherapy applications and compared its performance with Std‐FDK and Std‐iCBCT acquired on a previous generation TrueBeam v2.7 platform to assess whether similar benefits can be achieved using existing clinical infrastructure. To the best of our knowledge, this is the first study to directly compare the image quality, HU accuracy, and dose calculation performance of HyperSight iCBCT with both standard FDK and iterative CBCT reconstruction approaches in parallel.

### 4.1 HU to ED characterization

Results highlighted that reconstruction method has a substantial impact on HU accuracy with HS‐iCBCT demonstrating consistently lower ΔHU relative to baseline pCT values across both soft tissue and higher densities compared to Std‐iCBCT and Std‐FDK methods. On average, HS‐iCBCT had deviations within 7.7 ± 4.1 HU for *ρ* ≤ 1.08 g/cm^3^ and 33.5 ± 22.9 HU for higher densities up to *ρ* ≤ 1.92 g/cm^3^ for scanning parameters of 140 kV, 675 mAs. Std‐iCBCT yielded average deviations of 23.6 ± 13.5 HU and 46.2 ± 22.2 HU for the same density ranges whereas Std‐FDK showed the poorest agreement with pCT, with deviations of 38.0 ± 19.7 HU and 93.4 ± 57.6 HU. These findings are consistent with Washio et al^6^ who demonstrated that iCBCT reconstruction improved HU stability and dose calculation compared with FDK reconstruction in H&N cases, supporting the suitability of iterative reconstruction for CBCT‐based adaptive radiotherapy workflows.

Further, the present study demonstrated that the selection of a 120 kV pCT protocol as the HU‐ED reference standard (as opposed to the conventional 140 kV pCT protocol) improved agreement with CBCT‐derived HU values across all reconstruction methods investigated. It is well established that cone‐beam CT inherently exhibits higher HU (in particular bone) than fan‐beam planning CT with a same nominal kV energy, primarily attributed to different beam geometries (i.e. more scattering component in cone‐beam out of fan‐beam plane). In addition, although the HyperSight CBCT acquisitions were performed at 140 kV, the reconstructed HU values are influenced by the complete imaging chain, including detector response, scatter correction and reconstruction algorithm, rather than acquisition energy alone. These findings highlight the importance of experimentally validating the optimal HU–ED calibration curve for a given CBCT system rather than selecting a calibration based solely on nominal tube potential. These findings suggest that adoption of a single HU‐ED calibration curve for CBCT dose calculation across different anatomical sites is feasible and has been adopted for routine dose calculations at our institution, potentially simplifying adaptive radiotherapy workflows.

### 4.2 Image quality and CBCT dose calculation optimization

Figure 4 demonstrates both energy and reconstruction dependent differences in HU stability. While iterative reconstruction improved precision, HS‐iCBCT provided the best balance of accuracy and consistency, particularly at higher acquisition energies relative to Std‐FDK and Std‐iCBCT as seen by ΔHU distributions showing reduced systematic bias with respect to the pCT. Increasing CBCT acquisition energy resulted in narrower ΔHU distributions and reduced systematic offset for all reconstruction methods, attributed to reduced image noise and improved photon penetration at higher energies. Overall, HS‐iCBCT achieved the most favorable balance between HU fidelity and image quality at 140 kV acquisition settings with a peak HU shift of approximately +15 HU and a narrow soft‐tissue histogram width (FWHM  =  39 HU). The 140 kV acquisition was selected as a clinically practical, “one‐size‐fits‐all” calculation protocol to provide consistent HU accuracy across multiple anatomical sites without the need for site‐specific acquisition parameters. While higher tube potentials may reduce soft tissue contrast compared with lower‐energy acquisitions, the primary objective of this study was to optimize quantitative image accuracy for dose calculation rather than contouring performance which will be determined locally. These findings support the use of higher‐energy CBCT acquisition protocols combined with iterative reconstruction for CBCT‐based dose calculation and adaptive radiotherapy workflows requiring accurate HU representation. This is consistent with Lim et al.^5^ who highlighted the importance of acquisition protocol optimization, reporting larger HU variation in CBCTs acquired with low scanning parameters using the full‐fan technique and reduced suitability for dose calculation in adaptive radiotherapy.

### 4.3 CBCT dose calculation

To date, most studies evaluating CBCT‐based dose calculation and adaptive radiotherapy workflows have focused on O‐ring linac platforms, where improved imaging hardware and reconstruction techniques support enhanced HU accuracy.^10^, ^11^, ^12^, ^13^, ^14^ Conventional C‐arm systems have historically necessitated alternative strategies for CBCT dose calculation, including site‐specific HU calibration curves, deformable image registration, and density override approaches, with reported dose discrepancies of up to 2%.^15^, ^16^, ^17^ Few studies have directly evaluated the impact of evolving on‐board kV imaging hardware and reconstruction software on HU accuracy and dose calculation performance in conventional TrueBeam systems or assessed their suitability side by side for adaptive radiotherapy applications.

Quantitative DVH and gamma analysis in phantom studies across different anatomical sites demonstrated that HS‐iCBCT had the closest agreement with pCT dose distributions across all anatomical regions with an average dose difference of 0.6% to PTVD98% and a median gamma pass rate of 98.8 % at 2%/1 mm (10% threshold). Std‐iCBCT demonstrated an average dose difference of 1.4% to the same DVH metric and a median gamma pass rate of 98.7%. Std‐FDK reported the largest dose differences with an average difference of 1.6% to the PTV D98% and a median gamma pass of 96.0%. Differences were most pronounced in the lung phantom, where HS‐iCBCT maintained close agreement with pCT(< 0.5% to PTV_D99%_) despite low‐density heterogeneity increasing susceptibility to scatter artefacts and HU inaccuracies.

Decreasing to 1%/1 mm (10% threshold) resulted in median gamma pass rates of 98.0 % for HS‐iCBCT compared to 97.6% and 94.0% with Std‐iCBCT and Std‐FDK respectively. These results are comparable with previous results evaluating HyperSight for O‐ring platforms. Bogowicz et al^12^ reported agreement above 93.0% for 2%/1 mm for anthropomorphic phantoms representative of H&N, Brain, liver and lung anatomies. Further, Duan et al^11^ demonstrated that HyperSight iterative CBCT provided dose calculation accuracy comparable to synthetic CT for online adaptive radiotherapy workflows in pelvis and breast patients with median gamma pass rates of 99.4% at 2%/2 mm and 96.5% at 1%/1 mm with 10% threshold. The current study extends these observations to a conventional TrueBeam platform and demonstrates that many of the image quality improvements associated with HyperSight are retained outside dedicated O‐ring adaptive systems.

Although HS‐iCBCT demonstrated the lowest overall HU deviations and best dosimetric agreement, these improvements should be interpreted as the cumulative effect of multiple system enhancements rather than iterative reconstruction alone. Compared with previous‐generation TrueBeam 2.7 platform, the HyperSight v4.1 system incorporates a larger detector, improved scatter‐grid design, faster gantry rotation and updated iterative reconstruction software, all of which contribute to improve image quality and quantitative accuracy. In contrast, the comparison between Std‐iCBCT and Std‐FDK directly isolates the effect of iterative reconstruction independent of hardware changes. While differences between the two iterative reconstruction implementations were not consistently statistically significant following correction for multiple comparisons (*p* > 0.05). HS‐iCBCT demonstrated the clearest and most consistent improvement relative to Std‐FDK, suggesting that the combination of enhanced imaging hardware and iterative reconstruction software provides the greatest benefit for quantitative CBCT performance. Std‐iCBCT generally demonstrated intermediate performance between HS‐iCBCT and Std‐FDK; however, differences relative to Std‐FDK did not consistently reach statistical significance following post‐hoc analysis. Nevertheless, the observed trends suggest that iterative reconstruction alone may still provide improvements in image quality and HU consistency on conventional TrueBeam systems. This is clinically relevant for centers without access to dedicated adaptive O‐ring platforms or the resources required for major hardware upgrades, as optimization of iterative reconstruction workflows alone may still provide meaningful improvements in quantitative CBCT performance.

Several limitations of this study should be acknowledged. First, the analysis was performed using anthropomorphic phantom datasets rather than patient data, limiting direct assessment of anatomical variation, motion, and clinical workflow complexity. In addition, only four anatomical sites were evaluated, which may have limited the statistical power of the Friedman analysis and reduced sensitivity for detecting smaller inter‐method differences. Dose calculation accuracy was not evaluated in the presence of high‐density metal implants which may affect CBCT HU fidelity and image reconstruction accuracy, particularly for H&N anatomies, and could introduce additional uncertainty in CBCT‐based dose calculation. Further, residual registration uncertainty may have contributed to small dosimetric variations between datasets.

Future work should evaluate the robustness of HS‐iCBCT in larger patient cohorts and adaptive workflows, including contouring accuracy, dosimetric accuracy in the presence of metallic implants such as hip prostheses and dental implants, anatomical heterogeneity, and free‐breathing motion to further characterize the limitations of iterative CBCT reconstruction under challenging imaging conditions.

## 5 CONCLUSION

This study provides a comprehensive evaluation of image quality and dosimetric accuracy using HyperSight‐enabled iterative CBCT on TrueBeam platform relative to Std‐iCBCT and Std‐FDK reconstruction methods. Image quality and dosimetric accuracy were evaluated across multiple anatomical sites and optimized 140 kV CBCT acquisition protocols combined with application of a single baseline pCT HU–ED calibration curve proved feasible for CBCT‐based dose calculation. HS‐iCBCT demonstrated improved HU accuracy, reduced voxel‐wise HU variability, and superior dosimetric agreement with pCT compared with both standard reconstruction approaches, with the greatest improvements observed relative to Std‐FDK in heterogeneous thoracic anatomy. Modest improvements were observed relative to Std‐iCBCT, supporting the continued feasibility of Std‐iCBCT for dose verification workflows. These findings support the feasibility of HS‐iCBCT for CBCT‐based dose calculation and adaptive radiotherapy workflows on conventional TrueBeam platforms, with enhanced hardware and iterative reconstruction providing the optimal solution for substantial improvement in HU fidelity and dose calculation accuracy.

## AUTHOR CONTRIBUTIONS

**Niamh L Clarke**: Conceptualisation; methodology & data collection; formal analysis; software; investigation; visualisation; writing—original manuscript draft; writing—review & editing. **Jeremy L Hughes**: Software; visualisation; writing—review & editing. **Derrick Wanigaratne**: Visualisationn; writing—review & editing. **Adam U Yeo**: Conceptualisation; data collection & analysis; writing—review & editing.

## CONFLICT OF INTEREST STATEMENT

The authors declare no conflicts of interest.

## ETHICS STATEMENT

Ethics approval was not required for the phantom‐based component of this study, as no patient data were involved. The qualitative patient images presented in this study were included under existing departmental ethics approval for retrospective planning studies evaluating new techniques and technologies: Evaluation of Novel Radiotherapy Treatment Planning Techniques (Research No. 17_95R, Version 5, approved July 14, 2023).

## Data Availability

The data that support the findings of this study are available from the authors upon reasonable request.
